# Radiotherapy as a Contributing Factor to Carotid Vasculopathy Leading to Tandem Internal Carotid-Middle Cerebral Artery Occlusion Stroke

**DOI:** 10.7759/cureus.99464

**Published:** 2025-12-17

**Authors:** Abdalrahman Shaban, Kashif Musarrat

**Affiliations:** 1 Department of Medicine, University Hospitals of Leicester NHS Trust, Leicester, GBR; 2 Department of Neurology/Stroke Medicine, University Hospitals of Leicester NHS Trust, Leicester, GBR

**Keywords:** carotid stenosis, carotid vasculopathy, head and neck cancer, mechanical thrombectomy, tandem occlusion

## Abstract

A 64-year-old man with a history of p16-positive oropharyngeal squamous cell carcinoma treated with high-dose bilateral radiotherapy in 2017 presented with sudden-onset left-sided weakness, dysphasia, and homonymous hemianopia. Initial computed tomography and computed tomography angiography demonstrated complete right internal carotid artery (ICA) occlusion with a tandem right middle cerebral artery (M1) thrombus. Magnetic resonance imaging confirmed multiple acute infarctions within the right middle cerebral artery territory and watershed zones. He underwent urgent mechanical thrombectomy with angioplasty and stenting of the right ICA, achieving successful reperfusion. His medical history included hypertension, hypothyroidism, and previous smoking. This case highlights radiotherapy as a contributing factor to accelerated carotid vasculopathy and delayed large-vessel ischaemic stroke. It underscores the importance of long-term vascular surveillance in cancer survivors who previously received high-dose neck radiotherapy and demonstrates that mechanical thrombectomy with carotid stenting is a feasible treatment strategy in radiation-associated tandem vessel occlusion.

## Introduction

Radiotherapy plays a key role in improving survival for head and neck cancer, particularly in patients with locally advanced oropharyngeal squamous cell carcinoma. However, late vascular complications are increasingly recognised as survivorship improves. Radiation-induced vasculopathy can develop several years after treatment and may manifest as accelerated atherosclerosis, stenosis, or complete arterial occlusion [1-5].

These vascular changes arise due to endothelial injury, inflammation, and progressive fibrosis affecting large cervical vessels, most commonly the internal carotid arteries. Although the risk is amplified by conventional vascular factors such as hypertension and smoking, radiation remains an independent and significant contributor [1,6,7].

Radiation-associated carotid disease is under-recognised and may only present when patients experience a transient ischaemic attack or acute ischaemic stroke. Tandem occlusions, involving simultaneous extracranial ICA occlusion and intracranial vessel involvement, remain rare but clinically important as they require urgent interventional management.

This case highlights the importance of recognising delayed radiation-related vascular pathology and demonstrates that mechanical thrombectomy with carotid stenting can be an effective treatment strategy in selected patients.

In the present case, the patient presented with a high National Institutes of Health Stroke Scale (NIHSS) score on admission, reflecting severe neurological deficit [8,9].

## Case presentation

A 64-year-old man presented with sudden-onset left-sided weakness, facial asymmetry, hemianopia, and expressive dysphasia. On initial assessment, left arm and leg weakness was noted, with subsequent development of expressive dysphasia and homonymous hemianopia. His NIHSS score on arrival was 18 out of 42. His medical history included p16-positive tonsillar squamous cell carcinoma treated eight years earlier with bilateral high-dose radiotherapy (75 Gy delivered in 30 fractions) alongside weekly cisplatin. Additional comorbidities included hypertension, hypothyroidism, and a history of smoking.

**Figure 1:**
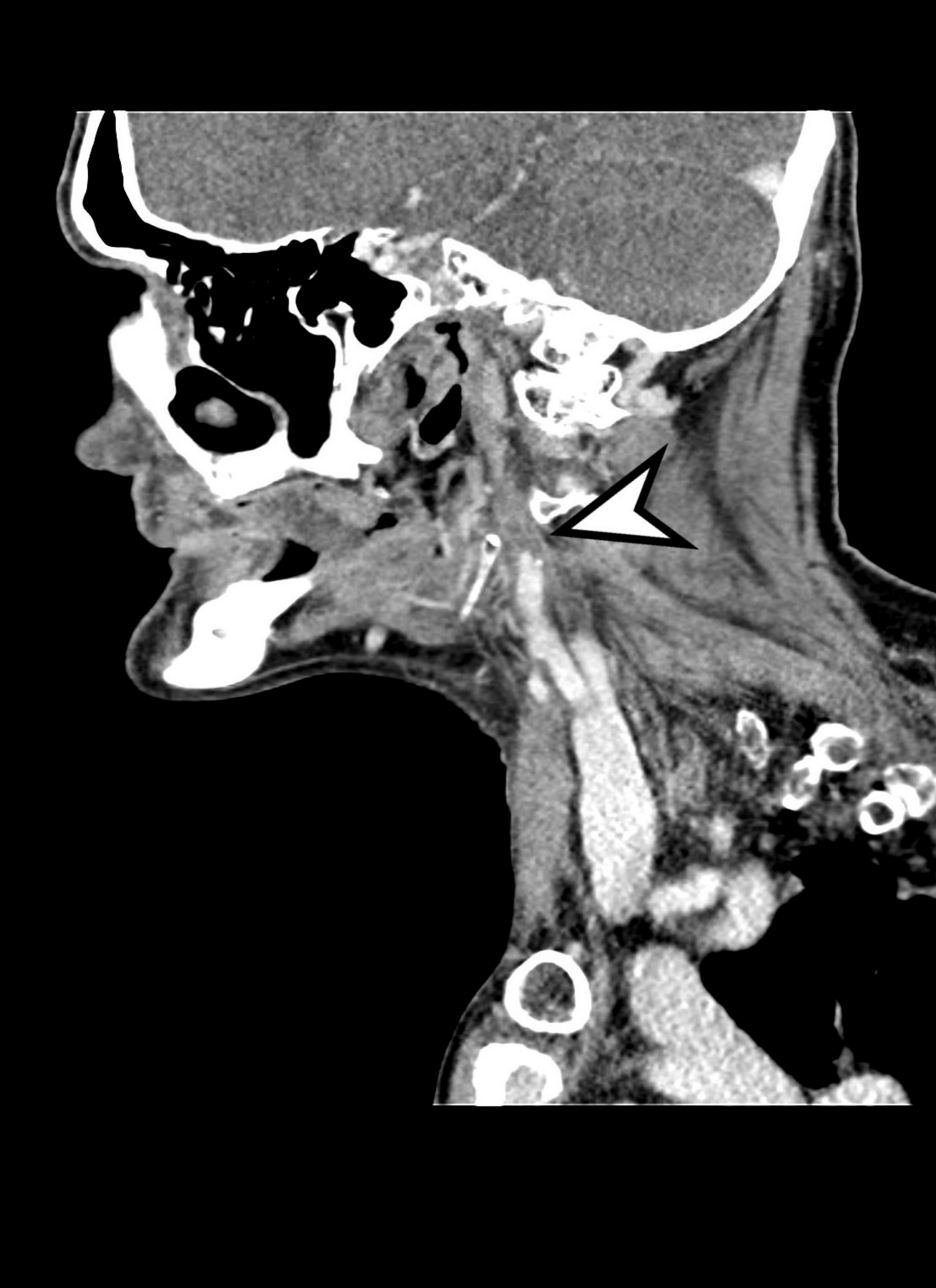
Sagittal reconstruction image from computed tomography angiography (CTA) demonstrating complete occlusion of the right cervical internal carotid artery (white arrowhead).

**Figure 2:**
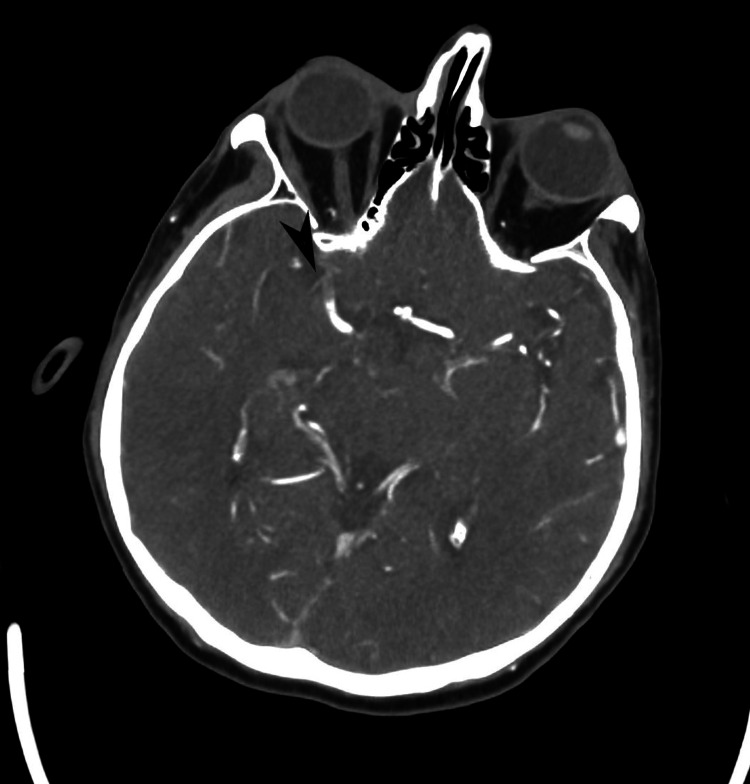
Axial image from subsequent computed tomography angiography (CTA) demonstrating a thrombus within the right middle cerebral artery (M1 segment) (black arrowhead).

**Figure 3:**
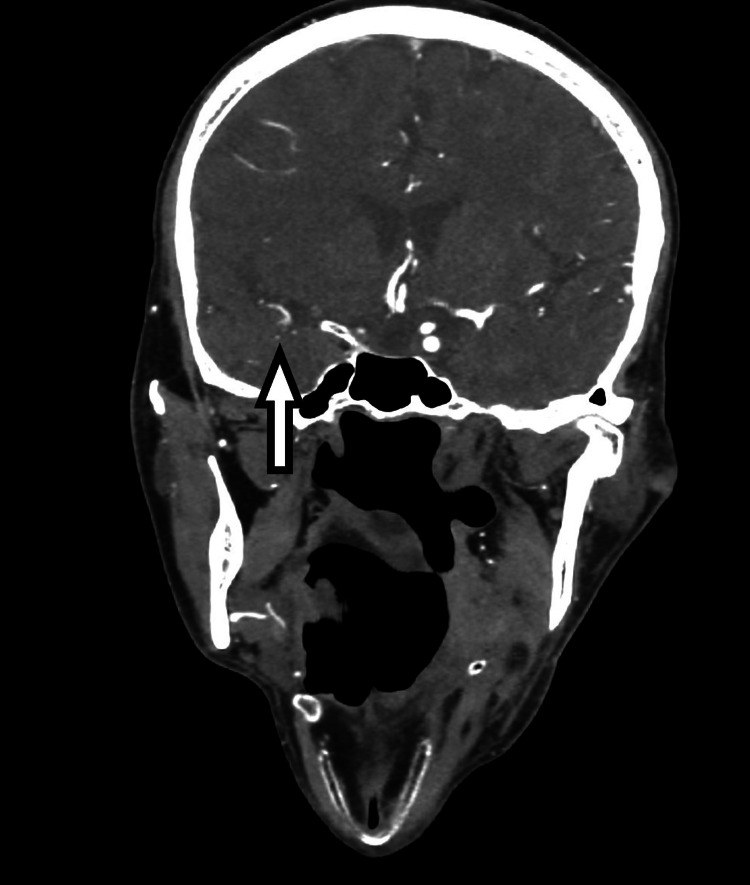
Coronal reconstruction image from computed tomography angiography (CTA) demonstrating tandem occlusion of the right middle cerebral artery M2 branches (white arrow).

Initial non-contrast computed tomography of the head revealed subtle right frontal hypodensity. Computed tomography angiography demonstrated complete occlusion of the right internal carotid artery (ICA) beginning shortly beyond its origin, with partial collateral reconstitution distally. A new filling defect was also identified within the right middle cerebral artery (M1 segment), with reduced opacification of downstream M2 branches, in keeping with tandem occlusion (Figures 1-3).

Additionally, duplex ultrasound of the contralateral internal carotid artery demonstrated approximately 60% stenosis with sonographic features possibly consistent with radiation-induced vascular change.

**Figure 4:**
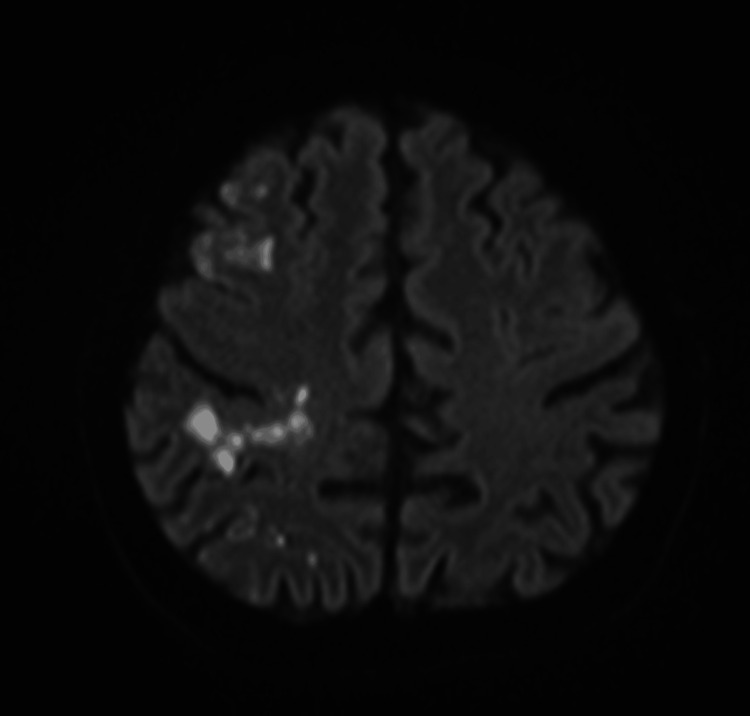
Magnetic resonance imaging diffusion-weighted imaging sequence demonstrating areas of restricted diffusion consistent with multiple acute ischemic infarctions in the right middle cerebral artery territory.

Magnetic resonance imaging confirmed multiple acute ischaemic infarcts involving the right middle cerebral artery territory and watershed zones (Figure 4).

As the patient’s neurological symptoms progressed, he was transferred to a regional neuroscience centre for urgent endovascular intervention. Mechanical thrombectomy was performed with subsequent right internal carotid artery stenting, resulting in satisfactory reperfusion.

Following rehabilitation, the patient demonstrated gradual neurological improvement. At discharge, he retained mild left-sided weakness and dysarthria but achieved functional independence.

## Discussion

Radiation-induced carotid vasculopathy is increasingly recognised as a late complication of head and neck radiotherapy, particularly in long-term cancer survivors. Vascular injury results from endothelial cell loss, intimal proliferation, inflammation, and progressive fibrosis, eventually leading to arterial stiffening and accelerated atherosclerosis [1,6,7]. These structural and functional changes can progress silently for years before manifesting clinically. The latency period typically ranges from five to fifteen years following treatment, consistent with the timeline in this case [2].

The condition may mimic conventional atherosclerotic disease; however, certain features raise suspicion for a radiation aetiology, including involvement distal to the carotid bulb, long-segment smooth narrowing, and bilateral vascular change corresponding to previous radiation fields. In this patient, previous radiotherapy with high-dose bilateral neck irradiation likely contributed to progressive carotid pathology, with conventional risk factors such as hypertension and smoking further compounding the process [1-3].

Tandem occlusion involving simultaneous extracranial carotid and intracranial large-vessel obstruction is uncommon but clinically significant due to the high risk of poor neurological outcome without timely intervention. Mechanical thrombectomy with or without carotid stenting has emerged as an effective strategy for selected patients with radiation-associated carotid artery disease [3]. In the present case, endovascular treatment resulted in successful reperfusion and favourable neurological recovery.

Previous reports have described radiation-induced carotid stenosis presenting with ischemic stroke; however, cases involving tandem extracranial-intracranial occlusion remain uncommon. Compared with prior case reports and small series, this case highlights a delayed presentation several years after radiotherapy with acute large-vessel occlusion requiring combined carotid stenting and mechanical thrombectomy. This case contributes additional clinical context supporting the use of endovascular intervention in carefully selected patients with radiation-associated carotid disease.

This case supports growing evidence that radiation-associated vasculopathy should be considered in stroke patients with a history of head and neck radiotherapy, even when the exposure occurred many years earlier. Earlier vascular screening strategies, such as carotid ultrasound in asymptomatic survivors, may enable timely detection and preventative management of significant stenosis before catastrophic events occur [3].

This report is limited by its single-patient design, and therefore the findings may not be generalisable to all patients with radiation-associated carotid vasculopathy.

## Conclusions

Radiation-induced carotid vasculopathy is an important late effect of head and neck radiotherapy and may present many years after treatment. This case demonstrates that radiation-associated vascular disease can lead to complex stroke patterns, including tandem occlusion requiring urgent endovascular intervention. Early recognition, awareness of this risk in cancer survivors, and consideration of surveillance imaging may reduce the likelihood of severe cerebrovascular events, as illustrated in this patient by the delayed, clinically silent progression of carotid disease following high-dose neck radiotherapy, before presentation with a large-vessel ischemic stroke. Clinicians should maintain a high index of suspicion when evaluating stroke in patients with a history of neck irradiation, even in the presence of traditional vascular risk factors.
